# Pulse waveform analysis in Zambian adults living with HIV on antiretroviral therapy: A cross‐sectional study of vascular dysfunction

**DOI:** 10.14814/phy2.70879

**Published:** 2026-04-17

**Authors:** Theresa Chikopela, Longa Kaluba, Shirley Mwaanga, Fastone M. Goma

**Affiliations:** ^1^ Department of Physiological Sciences, School of Medicine University of Zambia Lusaka Zambia

**Keywords:** arterial stiffness, HIV, pulse‐wave velocity, waveforms

## Abstract

People living with HIV (PLWH) experience a greater risk of cardiovascular disease due to HIV‐related vascular injury. Pulse waveform analysis can characterize arterial vascular dysfunction and this study compared arterial waveforms in PLWH on antiretroviral therapy (ART) with HIV‐negative controls. In this cross‐sectional study, participants were recruited from the University Teaching Hospitals, Lusaka (September 2018–June 2019). 55 PLWH on ART ≥2 years and 56 HIV‐negative controls were recruited. Anthropometry, body composition, haemodynamics, and pressure waveforms were recorded using the Complior Analyze unit with their corresponding pulse wave velocities. Waveforms were classified by augmentation‐index/pulse‐pressure ratio as Types A (≥0.12), B (0–0.12), or C (<0). Appropriate parametric and nonparametric tests were used for analysis. Analysis of 111 waveforms showed Type‐A predominance in PLWH (80% A, 3.6% B, 16.6% C) and HIV‐negative controls (57% A, 8.9% B, 33.9% C). Fisher's Exact Test indicated an association between waveform type and HIV status (*p* = 0.037). Multinomial logistic regression revealed that PLWH were more likely to exhibit Type‐A waveform than Type‐B or C (Wald *χ* ^2^(2) = 48.17, *p* < 0.001). HIV status is associated with a shift toward Type‐A waveform, reflecting altered arterial function. Pulse waveform analysis may serve as a useful non‐invasive tool for vascular risk profiling in PLWH.

## 1 INTRODUCTION

Cardiovascular disease (CVD) is a leading global cause of death and imposes a disproportionate burden on people living with HIV (PLWH), even when viral replication is suppressed (Freiberg et al., 2013; World Health Organization W, 2025). Traditional risk factors and antiretroviral therapy (ART) account for part of this risk, but evidence points to HIV‐related vascular injury and persistent endothelial dysfunction as central drivers of clinical events (Chikopela et al., 2021; Dolan et al., 2005; Friis‐Møller et al., 2007; Hsue et al., 2009; Lundgren et al., 2008). Sub‐Saharan Africa (SSA) accounts for two‐thirds of the 37.7 million people living with HIV globally (UNAIDS, 2025) with Zambia having a prevalence of 11% among adults aged 15 years and above (ZAMPHIA, 2025). CVD incidence in PLWH is approximately 1.5–2.0‐fold higher in high‐income cohorts (Triant et al., 2007) and is rising in SSA where the HIV burden is greatest (Rosenberg et al., 2023).

In studies of vascular injury characteristics, the measurement of arterial stiffness via pulse wave velocity (PWV) and the analysis of pulse waveforms have emerged as essential biomarkers of vascular dysfunction (Chikopela et al., 2021; Kaluba et al., 2021). As the compliance of vessels reduces, there is an increase in PWV detecting both central (carotid‐femoral PWV‐ cfPWV) and early peripheral alterations (carotid radial PWV – crPWV) (Ghosh et al., 2019; Laurent et al., 2006; Willum‐Hansen et al., 2006). The vascular pulse waveform rises to a peak in response to systole and is followed by a diastolic decline (Barrett & Ganong, 2018). The key measures of the waveforms include an augmentation pressure (AP) that is a pressure increment from the inflection (systolic shoulder, P1) to the peak of systole, as well as the augmentation index (AIx). The latter is AP expressed as a proportion of the pulse pressure.

The morphology of pulse waveforms includes waveforms A, B and C (Hughes et al., 2013; Ouyoung et al., 2022). Type A waveforms are characterized by an early systolic peak with a large positive augmentation (∆P/PP >0.12) (Mulder et al., 2022; Vlachopoulos et al., 2011). Physiologically, a forward wave is generated by the heart's contraction, while the reflected pressure waves move back toward the heart at the end of the systolic period. The forward wave can encounter regions of impedance mismatch, such as arterial branches or areas of increased stiffness, leading to partial wave reflection that occurs earlier than at the end of the systolic period. It then interacts with the forward wave, producing late systolic pressure augmentation (Boutouyrie et al., 2021). In the Type A waveform, the quick movement causes more work on the cardiovascular system due to the increased afterload on the ventricle as it superimposes on the forward wave at the inflection point. This then results in an augmentation of the systolic pressure and pulse pressure (Kim et al., 2021; Vasan, 2008). Type B waveforms have a later systolic peak with modest augmentation (0 < ∆P/PP <0.12), and Type C has a negative augmentation (∆P/PP <0.0) attributable to a pronounced decompression wave that reduces both pressure and flow (Hughes et al., 2013).

These waveform patterns are said to be clinically significant in populations with heightened CVD risk such as PLWH. Elevated PWV and more prevalent Type A waveform suggest an accelerated vascular dysfunction likely due to HIV‐mediated endothelial disruptions (Chikopela et al., 2021; Kaluba et al., 2023). HIV impairs nitric oxide bioavailability through viral proteins (gp120, Tat) and co‐receptors (CCR5) activity leading to compromised vascular compliance (Duvenhage et al., 2018). Owing to their reproducibility and sensitivity, pulse waveforms may serve as a valuable tool for subclinical vascular disease. The waveforms may also offer a means to explore how HIV‐related endothelial dysfunction may interact with body composition and CVD risk (Anderson, 2006; Cappuccio et al., 2008). While PWV has been studied in SSA HIV populations, detailed pulse waveform morphology classification using AIx/pulse‐pressure ratios remains largely unexplored in this region. To our knowledge, this is the first such study in Zambia with an approach that offers additional information beyond PWV alone in characterizing vascular dysfunction. Our primary aim was to evaluate arterial pulse waveforms in PLWH on ART compared with HIV‐negative controls.

## 2 MATERIALS AND METHODS

### 2.1 Study design and setting

We conducted a cross‐sectional analytical study comparing vascular function between PLWH on ART and HIV‐negative controls recruited at the University Teaching Hospitals (UTH), Lusaka, from September 2018 to June 2019. The participants were drawn from the NUSTART cohort, the Adult Infectious Disease Centre (AIDC), and the UTH outpatient department. A paper screening log recorded assessed individuals and eligible participants received unique study IDs. We included Lusaka residents aged 18–45 years; PLWH on ART ≥2 years with clinically presumed viral suppression; controls required a negative rapid HIV test. We excluded individuals with a clinical diagnosis of hypertension, diabetes mellitus, or respiratory disease, as well as pregnant women, smokers, and those with self‐reported infections that could influence PWV or other cardiovascular measurements.

The study used continuous response variables for PWV, comparing PLWH with matched control subjects. In a previous study (Giuseppe et al., 2008), the response among PLWH was normally distributed with a standard deviation of 1.21. Using these parameters, a true difference of 0.66 m/s in cfPWV between PLWH and controls would require 53 participants per group to achieve 80% power at a type I error probability of 0.05. Similar methodological adaptations have been applied in our earlier studies (Chikopela et al., 2021), supporting the robustness of this approach in comparable populations.

Informed consent was obtained from all participants. A baseline interview using the WHO STEPwise Approach to Surveillance (STEPS) instrument was used to capture sociodemographic, clinical data, and NCD risk factors. The WHO STEPS instrument is a standardized, validated tool developed for collecting NCD risk factor data across low‐ and middle‐income countries and has been widely applied across SSA. Anthropometry included height and weight measured with a Micro T3 PW‐BMI scale, waist and hip circumferences, and BMI. Body composition was assessed by Tanita‐305 (Tanita Corp., Tokyo, Japan) which uses bioelectrical impedance analysis after a ≥2‐h fast and bladder voiding, and by Bod Pod (Gold Standard Model 2007A; BOD POD COSMED Inc., Concord, CA, United States of America) air‐displacement plethysmography (software v5.2.0) in a controlled room with calibration before each session. Results were recorded and uploaded on RedCap (Vanderbilt University, Nashville, United States of America).

Waveforms, their specific characteristics (central systolic blood pressure, central pulse pressure, central diastolic blood pressure, augmentation pressure, end‐systolic blood pressure, timing of the systolic wave, timing of the reflected wave, left ventricular ejection time, diastolic time, first systolic peak, second systolic peak) data with the corresponding PWV and arterial stiffness index were obtained following the Complior protocol and acquired with the Complior Analyze unit V1.9 Beta Version 2013; ALAM‐Medical, Saint‐Quentin‐Fallavier, France. The Arterial Stiffness Index (ASI) is a composite measure of arterial stiffness derived from PWV, reflecting both the elastic and muscular components of the arterial wall. The carotid‐femoral ASI (cfASI) captures central arterial stiffness, while the carotid‐radial ASI (crASI) captures peripheral arterial stiffness. Both indices were calculated as the ratio of the distance between measurement sites (cm) to the transit time of the pulse wave (ms). Each PWV site was measured in triplicate at five‐minute intervals and the mean used as the final value. Representative waveforms for PLWH and HIV‐negative controls were identified and archived for detailed waveform morphology comparisons. Blood pressure and pulse rate were also measured in triplicate with an OMRON HEM‐757 in standing, sitting, and supine positions and each averaged, and two operators performed PWV readings for internal validation.

Ethical approval for the study was obtained from the University of Zambia Biomedical Research Ethics Committee (UNZABREC – IRB00001131 of IORG0000774) and Zambia's National Health Research Authority (NHRA). The study was conducted in accordance with the Declaration of Helsinki.

### 2.2 Data management and analysis

Data was uploaded to REDCap, de‐identified, password protected and later cleaned in Excel before analysis in Stata 15. Descriptive statistics included frequencies (%) for categorical data, mean (95% CI) for normally distributed continuous data, and median (IQR) for skewed data. Normality was assessed with QQ plots and the Shapiro–Wilk test. Between‐group comparisons were made using Student's *t*‐test or Wilcoxon rank‐sum as appropriate. To make categorical comparisons, Fisher's Exact, chi‐square, or two‐sample proportion tests were used as appropriate.

The classification of waveforms was done using AIx/pulse‐pressure ratios to define Types A (≥0.12), B (0–0.12), and C (<0) as described by Vlachopoulos, O'Rourke and Nichols (Vlachopoulos et al., 2011) and associations with HIV status were tested as described above. Multinomial logistic regression was used to assess the association between HIV status and waveform type. The waveform types were treated as a three‐level categorical outcome and HIV status as the predictor. Relative risk ratios (RRRs) and 95% confidence intervals were reported for each of the waveform types with one type designated as the reference outcome. In this analysis, waveforms Type A and C were used as references. This allowed for a simultaneous comparison of the likelihood of presenting with each waveform across the HIV status groups. Averaged Type A and C waveforms were constructed from normal weight PLWH and from HIV‐negative controls respectively with the assistance of Claude (Anthropic, San Francisco, CA, USA; claude.ai). Waveform digitisation, time‐normalization, and pressure rescaling to panel‐reported haemodynamic parameters were performed using Python scripts generated and executed within the Claude interface. All parameter values were calculated from data collected from the original Complior Analyze output prior.

## 3 RESULTS

Fifty‐five (55) PLWH and 56 HIV‐negative controls were recruited. PLWH had been on ART (efavirenz, emtricitabine, tenofovir DF) for over 2 years and were presumed virologically suppressed by clinical and CD4 criteria (James et al., 2013). Among the 55 PLWH, 20 (36%) were underweight, 19 (35%) normal weight, and 16 (29%) overweight. Baseline characteristics were mostly comparable, though PLWH were older (median 40 vs. 27 years), had a higher waist–hip ratio (0.81 vs. 0.798), heart rate (72 vs. 67 bpm), and pulse (79 vs. 73 bpm), while HIV‐negative controls had higher weight and systolic blood pressure as highlighted in Table 1.

**TABLE 1:** Demographic and clinical characteristics comparing PLWH and HIV‐negative controls.

Variable	PLWH	HIV‐negative controls	*p*
N	**55 (50%)**	**56 (50%)**	
Sex (female)	65%	67%	0.85
Age (years)	40 [20–45]	24 [18–43]	**<0.001** ***
Weight (kg)	54.8 [49.1–69.2]	60.5 [53.2–77.9]	**0.04** *
BMI (Tanita) (kg/m^2^)	20.4 [17.8–26.8]	22.8 [18.4–28.3]	0.11
Trunk fat (%)	20.1 [11.8–28.2]	21.2 [12.1–32.2]	0.66
Body fat percentage (%)	23.3 [15.0–31.7]	23.3 [14.1–34]	0.75
Total body fat mass (Tanita) (kg)	19 (11–26)	18 (14–21)	0.81
Total body fat mass (BodPod) (kg)	12.0 [9.2–25.5]	14.0 [6.6–29.2]	0.75
Waist‐hip ratio	0.81 [0.81–0.9]	0.798 [0.7–0.8]	**0.02** *
Heart rate (bpm)	72 (69–76)	67 (63–70)	**0.01** *
Central SBP (mmHg)	113 [103–121]	112 [102–124]	0.65
SBP (mmHg)	113 (109–117)	119 (115–124)	**0.048** *
DBP (mmHg)	80.0 [74–87]	79.8 [75–87]	0.84
Mean arterial pressure (mmHg)	93 [86–101]	90 [87–98]	0.48
Pulse (bpm)	79 (75–83)	73 (70–76)	**<0.001** ***
Alcohol consumption (yes)	30	24	0.16
cfPWV (m/s)	7.5 [6.1–9.2]	7.1 [6.0–8.1]	0.09
cfASI (cm/ms)	2.68 (2.45–2.91)	2.38 (2.26–2.50)	**0.03** *
crPWV (m/s)	10.2 (9.6–10.8)	9.7 (9.2–10.2)	0.20
crASI (cm/ms)	2.40 (2.25–2.55)	2.21 (2.10–2.32)	**0.02** *

*Note*: Values are in percentage, mean (95% confidence interval [95% CI]), and in case of skewed distribution of data, medians [interquartile ranges [IQR]]; value (number – number) are mean values.

Abbreviations: BMI, body mass index; crASI, carotid–radial arterial stiffness index; crPWV, carotid–radial pulse wave velocity; DBP, diastolic blood pressure; PLWH, people living with HIV; SBP, systolic blood pressure. Bold values indicate statistically significant differences between groups (*p* < 0.05).

* *p* < 0.05.

*** *p* < 0.001.

Further, Table 1 shows a significantly higher cfASI among PLWH with a mean cfASI of 2.68 cm/ms, 95% CI 2.45–2.91 compared to HIV‐negative controls (2.38 cm/ms 95% CI 2.26–2.50; *p* = 0.03), indicating increased central arterial stiffness. Similarly, crASI values were elevated among PLWH with a crASI of 2.40 cm/ms, 95% CI 2.25–2.55 compared to HIV‐negative controls (2.21 cm/ms 95% CI 2.10–2.32; *p* = 0.02), suggesting heightened peripheral arterial stiffness as well.

### 3.1 Pulse waveforms stratified by HIV group

A total of 111 carotid‐femoral waveforms were analyzed, comprising 55 waveforms from participants with HIV and 56 from the HIV‐negative controls.

#### 3.1.1 Typical representation waveforms from PLWH and HIV‐negative controls

**FIGURE 1:**
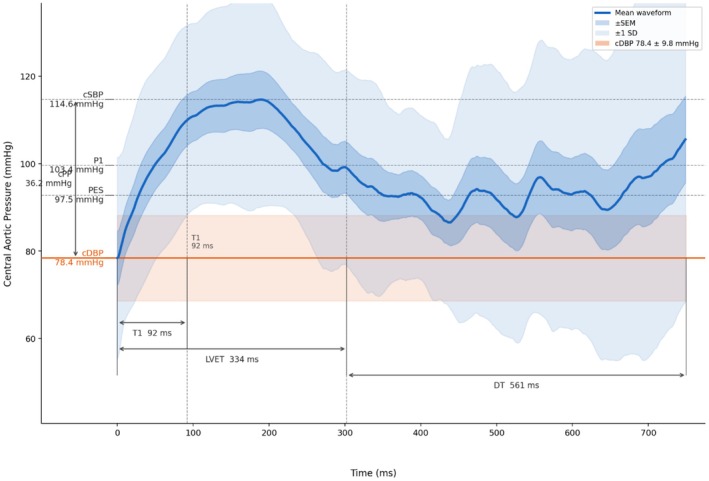
Averaged central aortic pressure Type A waveform from PLWH. Generated with the assistance of Claude (Anthropic, San Francisco, CA, USA). AP, augmentation pressure; cDBP, central diastolic blood pressure; cPP, central pulse pressure; cSBP, central systolic blood pressure; DT, diastolic time; LVET, left ventricular ejection time; P1, first systolic peak; P2, second systolic peak; PES, end‐systolic blood pressure; PLWH, people living with HIV; T1, timing of the reflected wave; Tsys, timing of the systolic wave.

Figure 1 represents an example of a typical Type A waveform that was predominant among PLWH.

The averaged waveform shows a steep, uninterrupted increase in pressure of the forward wave to its first peak (P1 = 103.4 ± 17.5 mmHg) from the foot of the wave, driven by left ventricular ejection. The reflected wave arrives during early systole, adding to the forward pressure wave and causing a further rise in systolic pressure to its peak (cSBP = 115.0 ± 16.8 mmHg), resulting in an augmentation pressure of 11.4 ± 5.4 mmHg. The AIx, as shown in Figure 1, is characterized by being above 0.25 (mean = 0.32 ± 0.15), indicating that the reflected wave arrives and augments the central aortic pressure during systole rather than diastole, consistent with increased arterial stiffness. The early return of the reflected wave, as evidenced by a short T1 of 92 ± 33.9 ms, is completed well before the end of left ventricular ejection (LVET = 334 ± 66.4 ms), resulting in an elevated systolic load on the left ventricle. The waveform also demonstrates a late systolic shoulder between the systolic peak and the incisura, followed by a modest pressure rise in early diastole, reflecting the characteristic pattern of premature wave reflection seen in conditions associated with arterial stiffening.

**FIGURE 2:**
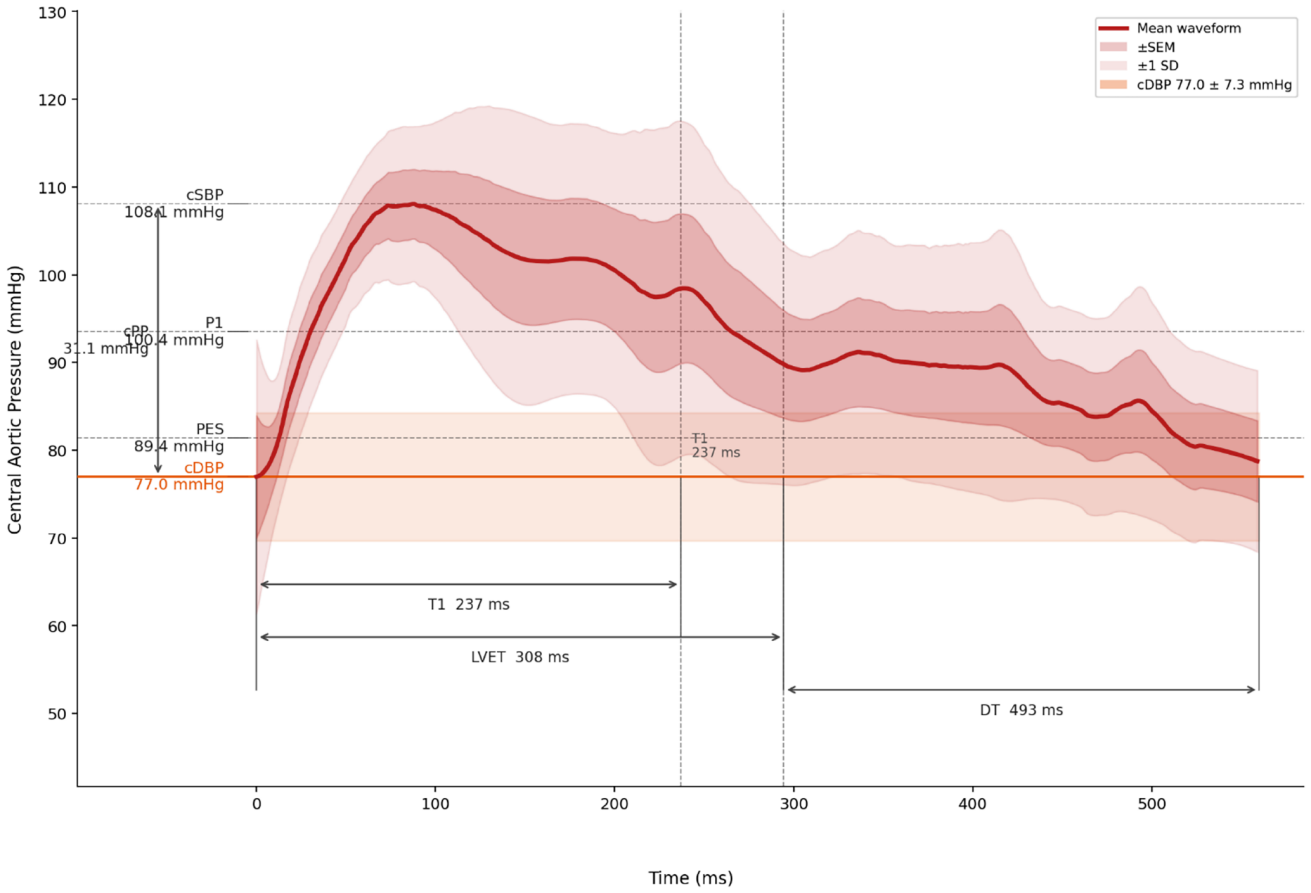
Averaged central aortic pressure Type C waveform from the HIV‐negative control group. Generated with the assistance of Claude (Anthropic, San Francisco, CA, USA). AP, augmentation pressure; cDBP, central diastolic blood pressure; cPP, central pulse pressure; cSBP, central systolic blood pressure; DT, diastolic time; LVET, left ventricular ejection time; P1, first systolic peak; P2, second systolic peak; PES, end‐systolic blood pressure; PLWH, people living with HIV; T1, timing of the reflected wave; Tsys, timing of the systolic wave.

The HIV‐negative controls demonstrated a numerically higher proportion of Type C waveforms, with Figure 2 showing the representative averaged waveform from this cohort. The waveform was characterized by a steep, uninterrupted increase in pressure of the forward wave to its first systolic peak (P1 = 100.4 ± 10.2 mmHg) from the foot of the wave, driven by left ventricular ejection. The reflected wave added to the forward pressure wave in late systole or early diastole, consistent with the later return of the reflected wave relative to systole. The AIx was characterized as being less than 0.0 (mean = −0.24 ± 0.17) as shown in Figure 2, indicating that the peak systolic pressure is generated by the forward wave alone, with the reflected wave arriving after the systolic peak and therefore not augmenting systolic pressure. The timing of the reflected wave was completed after the systolic wave had elapsed, as evidenced by the longer T1 of 237 ± 56.4 ms relative to LVET of 308 ± 25.9 ms. The waveform also shows a late systolic shoulder between the first peak and the incisura, and a second pressure rise in early diastole, consistent with the characteristic pattern of late wave reflection seen in individuals with preserved arterial compliance. Supplementary Table S1 shows the means and SD for each variable for both Type A and C waveforms in PLWH and HIV‐negative controls respectively.

#### 3.1.2 Waveform differences in PLWH and HIV‐negative controls

A two‐way contingency table analysis was conducted to evaluate whether type of waveform was associated with HIV status. The two variables were waveform with three levels (Type A, B and C) and HIV status with two levels (PLWH and HIV‐negative controls). A Fisher's Exact Test showed a statistically significant relation between type of waveform and HIV status *p* = 0.037 as shown in Table 2.

**TABLE 2:** Waveform Types in PLWH and HIV‐negative controls.

Waveform types	PLWH (*n* = 55)	HIV‐negative controls (*n* = 56)	*p* value
Type A	44 (80%)	32 (57%)	**0.014** *
Type B	2 (4%)	5 (9%)	0.438
Type C	9 (16%)	19 (34%)	**0.048** *
Overall			**0.037** *

*Note:* Bold values indicate statistically significant differences between groups (*p* < 0.05).

Abbreviation: PLWH, people living with HIV.

* *p* <0.05.

Subsequent pairwise analyses showed that the difference was driven by the distribution of waveform Types A and C between PLWH and HIV‐negative controls. There were more Type A waveforms among PLWH (*p* = 0.014) and a significantly higher number of Type C waveforms in the HIV‐negative controls (*p* = 0.048).

We further examined the association between HIV status and waveform types using multinomial logistic regression, specifying Type C waveform as the base outcome. The model was fitted with 111 observations and demonstrated good overall fit (Wald *χ* ^2^ = 48.17, *p* < 0.001). The relative risk ratios (RRRs) indicated that individuals living with HIV had increased odds of being classified in category A or B relative to having a Type C waveform. Specifically, PLWH were over two times more likely to have a Type A waveform than Type C waveform (RRR = 2.05; 95% CI: 1.53–2.75; *p* < 0.001). PLWH were significantly less likely to present with a Type B waveform than a Type C waveform (RRR = 0.37; 95% CI: 0.20–0.70; *p* = 0.002).

We ran the regression again using Type A waveform as the reference category. The model was statistically significant (Wald *χ* ^2^(2) = 48.17, *p* < 0.001), indicating that HIV status significantly influenced waveform classification. Compared to individuals with Type A waveform, PLWH had significantly lower relative odds of exhibiting Type B waveform (RRR = 0.18, 95% CI: 0.10–0.33, *p* < 0.001) and Type C waveform (RRR = 0.49, 95% CI: 0.36–0.66, *p* < 0.001). These results suggest that PLWH are more likely to present with Type A waveform than with either Type B waveform or Type C, reinforcing a potential shift in waveform distribution associated with HIV status.

Table 3 shows a summary of the characteristics of the waveforms among PLWH and the HIV‐negative controls. There was no significant difference in the median values of the waveform characteristics between the HIV groups. This was except for central pulse pressure which was significantly different (*p* = 0.03) between groups for Type C waveform. PLWH had significantly lower central pulse pressure 30 mmHg; 95% CI = 29–37 compared to the HIV‐negative controls 40 mmHg; 95% CI = 31–53.

**TABLE 3:** Waveform characteristics comparing PLWH and HIV‐negative controls.

Waveform	PLWH (*n* = 55)	HIV‐negative controls (*n* = 56)	*p* value
Augmentation index			
Type A	19.6 [11.4–28.8]	25.6 [16.1–39.5]	0.07
Type B	1.6 [0.8–2.5]	1.7 [1.2–3.5]	0.44
Type C	−10.6 [−19.3 to −8.6]	−17 [−24 to −5.9]	0.66
Central systolic blood pressure (mmHg)			
Type A	114 [104–125]	111 [107–128]	0.59
Type B	98 [94–102]	100 [96–123]	0.70
Type C	104 [101–116]	114 [105–134]	0.10
Central diastolic blood pressure (mmHg)			
Type A	79 [74–85]	76 [74–85]	0.74
Type B	70 [69–70]	71 [70–75]	0.33
Type C	80 [73–86]	72 [69–79]	0.12
Central pulse pressure (mmHg)			
Type A	33 [28–41]	35 [27–42]	0.88
Type B	29 [25–32]	41 [22–41]	0.70
Type C	30 [29–37]	40 [31–53]	**0.03** *
First systolic peak (mmHg)			
Type A	109 [96–116]	104 [96–112]	0.52
Type B	98 [94–101]	99 [93–116]	1.00
Type C	99 [92–115]	105 [99–123]	0.43
Timing of reflected wave (ms)			
Type A	105 [76–137]	97 [67–115]	0.25
Type B	141 [125–156]	155 [128–159]	0.70
Type C	220 [176–229]	216 [185–229]	0.67
Timing of systolic wave (ms)			
Type A	211 [166–236]	216 [188–234]	0.56
Type B	228 [201–255]	184 [164–245]	0.44
Type C	159 [123–174]	109 [92–150]	0.11
Augmentation pressure (mmHg)			
Type A	7.5 [2.3–12.5]	9 [7–13]	0.24
Type B	0.5 [0–1]	3.0 [1–6]	0.24
Type C	6 [2–12]	9 [5–13]	0.46

*Note*: *Values shown as medians interquartile ranges [IQR]; PLWH, people living with HIV. Bold values indicate statistically significant differences between groups (*p* < 0.05).

* *p* <0.05.

## 4 DISCUSSION

The PLWH had higher crASI and cfASI with predominant Type A waveform, characteristic of arterial stiffness. This supported the idea that increase in arterial stiffness is indeed playing a role in the increase in CVDs among PLWH in Zambia. Among other risk factors of CVDs, variations in BMI have also been cited (Chiluba et al., 2017). This study found a significant association between HIV status and arterial waveform type, with PLWH on ART more likely to have a Type A waveform than Type B or C. Multinomial logistic regression confirmed that PLWH had over twice the odds of presenting with Type A waveforms compared to Type C, suggesting a shift in waveform distribution linked to HIV status. This pattern reflects increased arterial stiffness and altered vascular function in PLWH, reinforcing the use of waveform classification as a non‐invasive marker of vascular dysfunction.

Our findings are consistent with prior studies that have identified Type A waveforms as indicative of increased arterial stiffness and early systolic pressure augmentation. Both Murgo et al. (1980) and Hughes et al. (2013) described this waveform as a marker of arterial pathology and that its peak systolic pressure that occurs in early systole after a sharp inflection point causes increased pressure on the heart, augmenting the pressure that the left ventricle must work against. It has been reported in situations of increased arterial stiffness among pregnant hypertensives (Kaluba & Goma, 2020). This observed rise in AIx may reflect a decline in endothelial function in smaller, pre‐resistance arteries (Hughes et al., 2013; Schwartz et al., 2010).

Our findings contrast those of Lazar, Wu, Shi and Kagame A (Lazar et al., 2009), who found that HIV infection itself is not associated with greater arterial waveform reflection in Rwandan women. This discrepancy may be due to differences in population characteristics with our population including men, ART duration, and measurement techniques which were based on the radial artery measurements. AIx was similar between the HIV‐negative controls and PLWH, as was observed between HIV‐negative controls and non‐treated PLWH in South Africa by Kofoworola, Rajiv, Abolade and Ambrose (Kofoworola et al., 2015).

It is evident that AIx could be a plausible surrogate marker of arterial stiffness as noted in this study among others (Anderson, 2006; Kaluba et al., 2015). To further note the plausibility of this marker, both crASI and cfASI were used in which we observed that the PLWH had significantly higher cfASI and crASI compared to the HIV‐negative controls. This shows a higher composite measure of elastic plus muscular arterial stiffness and wave reflection (McEniery et al., 2006).

In characterizing the waveforms, no significant differences were noted in cSBP, cDBP, augmentation pressure, timing of the systolic wave, first systolic peak, or timing of the reflected wave in the two groups of interest except in central pulse pressure. It was significantly lower among PLWH compared to the control, suggesting a subclinical cardiovascular alteration that implies early ventricular‐arterial uncoupling or reduced cardiac output. This finding is consistent with prior reports in both HIV‐positive and general cardiovascular disease populations (Martínez‐Ayala et al., 2020).

Strengths of this study include the use of HIV‐negative controls and detailed waveform classification using AIx/pulse pressure ratios. However, the assumption of viral suppression without direct viral load measurement may have introduced misclassification bias. This assumption was based on the length the participants had been on ART and absence of clinical disease progression (James et al., 2013). The effects of ART could not be highlighted as the study was not powered to detect such differences. Further, given observed beneficial effects of ART on vascular function (Torriani et al., 2008; van Vonderen et al., 2009), the role that ART played in the present cohort could not be determined. The control group, partly recruited from outpatient clinics, may have included individuals with subclinical conditions affecting vascular function. A key limitation is the significant age difference between groups, with PLWH being approximately 13–16 years older than HIV‐negative controls. Age is a well‐established independent predictor of arterial stiffness and waveform morphology, and it is plausible that some or all the observed differences in waveform type distribution reflect this age disparity rather than HIV status. Future studies should aim to recruit age‐matched controls or perform age‐adjusted analyses to disentangle these effects. However, these findings can serve as preliminary characterisations of these variables in PLWH.

In conclusion, HIV status was significantly associated with a shift toward Type A waveform, reflecting increased arterial stiffness in PLWH on ART. Pulse waveform analysis offers a promising, non‐invasive tool for vascular risk profiling in this population and may help guide early interventions to mitigate cardiovascular risk.

## AUTHOR CONTRIBUTIONS

**Chikopela Theresa:** Conceptualization; data curation; formal analysis; funding acquisition; investigation; methodology; project administration; resources; software; supervision; visualization. **Longa Kaluba:** Investigation; project administration. **Shirley Mwaanga:** Validation. **Fastone M. Goma:** Conceptualization; methodology; supervision; validation.

## FUNDING INFORMATION

This work was supported by the Fogarty International Centre of the U.S. National Institutes of Health (NIH) under award number D43 TW009744, NIAID grant K23 AI100700, the NIH‐funded Vanderbilt Clinical and Translational Science Award from NCRR/NIH grant UL1 RR024975, the NIH‐funded Tennessee Centre for AIDS Research grant P30 AI110527 and NIH grant K01HL130497.

## CONFLICT OF INTEREST STATEMENT

The authors declare that they have no financial or personal relationships that may have inappropriately influenced them in writing this article.

## ETHICS STATEMENT

Ethical approval for the study was obtained from the University of Zambia Biomedical Research Ethics Committee (UNZABREC – IRB00001131 of IORG0000774) and Zambia's National Health Research Authority (NHRA). The study was conducted in accordance with the Declaration of Helsinki. Written informed consent for the use and publication of individual‐level physiological data was obtained as part of the original study consent process.

## DIVERSITY AND INCLUSION STATEMENT

In this study we addressed an underrepresented population and ensured equal representation across gender, age, and socioeconomic backgrounds. Authorship and capacity building involved males and females and different ranks of researchers from professors to medical students. We ensured equitable representation, inclusive collaboration, and capacity‐building.

## Supporting information


**Table S1.** Central Aortic Pressure Waveform Parameters: Type A vs. Type C.

## Data Availability

The authors confirm that the data supporting the findings of this study are available from the corresponding author upon reasonable request.
