# Supplementary material for: Pulse waveform analysis in Zambian adults living with HIV on antiretroviral therapy: A cross‐sectional study of vascular dysfunction
Source: Physiol Rep. 2026 Apr 17;14(8):e70879. doi: 10.14814/phy2.70879 (PMC13090532; doi:10.14814/phy2.70879)
Supplement: Supplementary file 1 — Table S1. Central Aortic Pressure Waveform Parameters: Type A vs. Type C. [file PHY2-14-e70879-s001.docx]

**SUPPLEMENTARY MATERIAL**

**Table 1: Central Aortic Pressure Waveform Parameters: Type A vs Type C**

| **Variable** | **Type A Waveform (PLWH, n = 17)** | **Type C Waveform (HIV-negative controls , n = 7)** |
| --- | --- | --- |
| cSBP (mmHg) | 114.6 ± 16.8 | 108.1 ± 8.4 |
| cDBP (mmHg) | 78.4 ± 9.8 | 77.0 ± 7.3 |
| cPP (mmHg) | 36.2 ± 10.1 | 31.1 ± 6.0 |
| P1 (mmHg) | 103.4 ± 17.5 | 100.4 ± 10.2 |
| PES (mmHg) | 97.5 ± 13.3 | 89.4 ± 10.9 |
| AP (mmHg) | 11.4 ± 5.4 | 7.7 ± 6.2 |
| AIx (%) | 32.3 ± 14.8 | -23.5 ± 16.9 |
| LVET (ms) | 333.8 ± 66.4 | 307.9 ± 25.9 |
| DT (ms) | 561.5 ± 134.6 | 493.1 ± 106.9 |
| T1 (ms) | 91.9 ± 33.9 | 237.0 ± 56.4 |
| HR (bpm) | 69.1 ± 8.6 | 75.0 ± 10.7 |

^cSBP= central systolic blood pressure, cPP=central pulse pressure, cDBP=central diastolic blood pressure, AP=augmentation pressure, PES= end-systolic blood pressure, Tsys=timing of the systolic wave, T1= timing of the reflected wave, LVET= left ventricular ejection time, DT= diastolic time, P1= first systolic peak, P2=second systolic peak; PLWH, people living with HIV.^
